# Device indication for calcified coronary lesions based on coronary imaging findings

**DOI:** 10.1007/s12928-023-00914-1

**Published:** 2023-02-13

**Authors:** Yuji Ikari, Shigeru Saito, Shigeru Nakamura, Yoshisato Shibata, Seiji Yamazaki, Yutaka Tanaka, Junya Ako, Hiroyoshi Yokoi, Yoshio Kobayashi, Ken Kozuma

**Affiliations:** 1 Department of Cardiology, Tokai University, Isehara, Japan; 2 Heart Center, Shonan Kamakura General Hospital, Kamakura, Japan; 3 Department of Cardiology, Kyoto Katsura Hospital, Kyoto, Japan; 4 Department of Cardiology, Miyazaki Medical Association Hospital, Miyazaki, Japan; 5 Department of Cardiology, Sapporo Higashi Tokushukai Hospital, Sapporo, Japan; 6 Department of Cardiology, Kitasato University, Tokyo, Japan; 7 Department of Cardiology, Fukuoka Sanno Hospital, Fukuoka, Japan; 8 Department of Cardiovascular Medicine, Chiba University Graduate School of Medicine, Chiba, Japan; 9 Department of Cardiology, Teikyo University, Tokyo, Japan

**Keywords:** Coronary calcification, Intravascular lithotripsy, Rotational atherectomy, Orbital atherectomy, Intravascular ultrasound, Optical coherence tomography

## Abstract

Performing percutaneous coronary intervention (PCI) for calcified lesions is still a major challenge. Calcified lesions are a cause of inadequate dilatation, leading to poor short- and long-term PCI outcomes. It has been suggested that modification for calcification before stent implantation might improve outcomes by providing adequate dilation. Intravascular lithotripsy (IVL) is available under insurance reimbursement in December 2022 in Japan. IVL is one candidate for a treatment device to modify calcified lesions in addition to atherectomy, such as rotational and orbital atherectomy, and special balloons, such as scoring and cutting balloons. Although the evidence for the indications, of these devices is insufficient, they must be used appropriately in clinical practice. In this report, we propose a method for determining an indication of these devices solely as per the coronary imaging findings with intravascular ultrasound or optical coherent tomography. This consensus document represents the collective opinion of experts on the best current indications and should be changed based on future evidence. However, we believe that it represents the optimal criteria for selecting treatment options in the current situation.

## Introduction

The treatment options for calcified lesions have increased with the reimbursement of intravascular lithotripsy (IVL). The method of appropriately selecting treatment devices for calcified lesions is unclear; therefore, in this report, we have proposed a method of selecting their indications by assessment with coronary imaging devices, like intravascular ultrasound (IVUS) or optical coherent tomography (OCT)/optical frequency domain imaging (OFDI). In Japan, coronary imaging is used in over 90% of all PCI cases. Additionally, the choice of device for treatment based on these findings is logical and can be indicated in almost all cases.

## Device selection strategy for calcified lesions

**Fig. 1:**
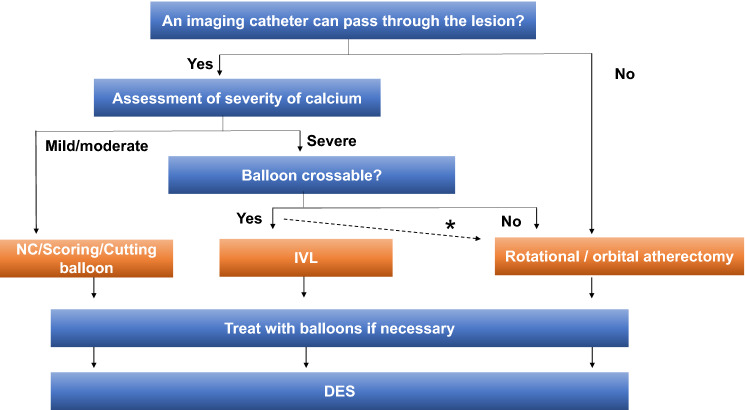
Device selection strategy for calcified lesions. *When rotational/orbital atherectomy is considered appropriate. *NC* non-compliant, *IVL* intravascular lithotripsy, *DES* drug-eluting stent

Step 1. The first attempt should be to pass the lesion with an imaging device, like IVUS or OCT/OFDI, after the guidewire passage (Fig. 1).

Step 2. If the imaging device passes through the lesion, the calcium severity should be assessed. Table 1 shows the method to assess the severity of calcium by IVUS or by OCT/OFDI. If the imaging device cannot pass through the lesion, rotational or orbital atherectomy should be considered.

**Table 1:** Calcium scoring by OCT/OFDI or by IVUS

OCT/OFDI	Points
Maximum calcium angle > 180°	2
Maximum calcium thickness > 0.5 mm	1
Calcium length > 5 mm	1
When the sum of the points is 3 or more, severe calcification is determined	

IVUS	Points
Superficial calcium angle > 270°and longer than 5 mm	1
360° of superficial calcium	1
Calcified nodule	1
Vessel diameter < 3.5 mm at the calcified lesion	1
When the sum of the points is 3 or more, severe calcification is determined	

Step 3. In case of severe calcium, an attempt to pass a balloon catheter should be made. If the balloon catheter passes the lesion, IVL should be selected to treat the calcified lesion. If the balloon catheter cannot pass the lesion, rotational or orbital atherectomy should be selected to treat the calcified lesion.

Step 4. In case of mild/moderate calcium based on IVUS or OCT/OFDI, non-compliant, scoring or cutting balloons should be selected to treat the calcified lesion.

Step 5. After performing calcium modification, the lesion should be dilated with balloons, if necessary. The lesions can be treated by drug-eluting stent implantation.

## Assessment of calcification

The severity of calcification is assessed by IVUS or OCT/OFDI. Angiographic findings are not used to assess the severity of calcification. Although there are various considerations, the evaluation method shown in Table 1 was adopted in this document [1, 2].

## Discussion

Even with advances in treatment devices, calcified lesions remain difficult targets while performing PCI. First, the passage of treatment devices might be difficult for calcified lesions. Second, stent dilatation might be inadequate, resulting in poor short- and long-term outcomes. Appropriate calcification modification before stent implantation might solve these problems.

Rotational atherectomy (rotablator) is a specific device that has been used for treating calcification in Japan for a long time since its reimbursement in 1997. Although restrictions were placed on the facilities, these were revised in 2020 to allow wider use [3]. Orbital atherectomy (diamondback) was reimbursed in 2017 in Japan and could be used to cut both when pulling and pushing calcification. IVL is a treatment device that generates shock waves from a balloon and was reimbursed in 2022. Balloons with mechanical resection, such as non-compliant (NC) balloons, scoring balloons or cutting balloons might be effective for mild or moderate calcification.

Although these calcification treatment devices are now available in Japan, there is still insufficient evidence for their indications. In this document, we proposed a new indication strategy for calcified lesions. This is noteworthy in that the indication is determined based on coronary imaging findings. This consensus document represents the collective opinion of the experts on the best current indications. However, the limitation of this report is that these expert opinions are not based on sufficient evidence. With the accumulation of further data, better indications should be considered in the future.

## Data Availability

Data is available.
